# Concerns on modeling postmenopausal osteoporosis on young female rats

**DOI:** 10.1186/s13018-019-1313-8

**Published:** 2019-12-18

**Authors:** Pavlos Lelovas, Ismene Dontas

**Affiliations:** Laboratory for Research of the Musculoskeletal System, School of Medicine, National & Kapodistrian University of Athens, 10, Athinas Str., K.A.T. Hospital, 145 61 Kifissia, Greece

Dear Editor,

We have read the research article entitled “A multi-factorial analysis of bone morphology and fracture strength of rat femur in response to ovariectomy” by Rocabado JMR, Kaku M, Nozaki K, Ida T, Kitami M, Ayogi Y, and Uoshima K which was published in the *Journal of Orthopaedic Surgery and Research* (2018;13:318). We would like to congratulate the authors for their diligent study but we would also like to pinpoint some of our concerns regarding the paper.

One of the first concerns is the selection of the rats’ age. Although the authors stated from the beginning of the article that their main focus is the study of postmenopausal osteoporosis, the age of the animals was 10 weeks old. According to Turner et al., “The growing rat is a useful model for evaluating the effects of endocrine, nutritional and other environmental factors on peak bone mass. The young rapidly growing rat is appropriate for studies designed to investigate factors related to peak bone mass but is a poor model for the adult human skeleton because skeletal growth is mediated by cellular processes which are not active in adults” [1]. Additionally, according to Jee and Yao, “it is reasonable to recommence the evaluation of treatment in the 9-month-old ovariectomized female rat. A female rat of this age has reached peak bone mass and can be manipulated to simulate clinical findings of post-menopausal osteoporosis.” [2]. Therefore, it is apparent that the selection of young growing rats, instead of mature ones, may not lead to accurate findings that could be translated to human postmenopausal osteoporosis.

As far as the conservation of bone specimens for biomechanical testing is concerned, Turner and Burr have reported regarding formaldehyde fixation of bone “Fixation in this manner increases collagen crosslinking and therefore will alter the properties of the bone tissue more significantly than alcohol preservation. Testing fixed samples only provides data relative to other fixed samples and should never be used as an accurate measure of the true properties of bone” [3]. Therefore, the article’s between groups comparisons are valid; however, their metric results do not represent true bone biomechanical parameters. The authors could have chosen to preserve their bone specimens in gauze soaked in normal saline and stored at − 20 °C, as supported by Turner and Burr [3].

Another point of concern was the short period for the establishment of osteoporosis (8 weeks). According to the literature, this is a very short time period to observe changes in bone strength. For the femoral mid-shaft, the earliest time after ovariectomy in order to observe decreases in biomechanical strength is 270 days, while in the first 3 months (12 weeks approximately), it is well documented that a transient increase in bone strength may be observed [2]. For this reason, the increase in bone strength observed by the authors is not surprising or contradictory as they stated but rather anticipated.

Furthermore, due to differences in bone physiology between humans and rats (lack of a well-developed system of Haversian remodeling, bone periosteal apposition occurring after ovariectomy in order to compensate endosteal resorption), Jee and Yao supported that “Since the most sensitive index of cortical bone loss involves the enlargement of the marrow cavity from the resorption of endocortical bone adjacent to marrow, a measurement of the thickness of the inner 1/2 or 1/3 of the cortex adjacent to the marrow proves to be meaningful” [2]. These changes may take longer time than 8 weeks to be observed [4]. Consequently, a longer experimental time for the establishment of bone loss in cortical bone, as well as appropriate methodology (evaluation of the inner instead of the full section of the femoral midshaft), may have led the authors to results more consistent with the existing literature and applicable to the human condition.

## Data Availability

Not applicable
